# Dental caries in the fossil record: a window to the evolution of dietary plasticity in an extinct bear

**DOI:** 10.1038/s41598-017-18116-0

**Published:** 2017-12-19

**Authors:** Borja Figueirido, Alejandro Pérez-Ramos, Blaine W. Schubert, Francisco Serrano, Aisling B. Farrell, Francisco J. Pastor, Aline A. Neves, Alejandro Romero

**Affiliations:** 1 Departamento de Ecología y Geología, Facultad de Ciencias, Universidad de Málaga, Málaga, 29071 Spain; 2 Center of Excellence in Paleontology and Department of Geosciences, East Tennessee State University, Johnson City, TN 37614 USA; 3 Natural History Museum of Los Angeles County, Dinosaur Institute, Los Angeles, CA 90007 USA; 4 La Brea Tar Pits and Museum, Los Angeles, CA 90036 USA; 5 Departamento de Anatomía y Radiología, Universidad de Valladolid, Valladolid, 47005 Spain; 6 Departamento de Odontopediatria e Ortodontia, Universidade Federal do Rio de Janeiro, Faculdade de Odontologia, Rio De Janeiro, 21.941-902 RJ Brazil; 7 Departamento de Biotecnología, Facultad de Ciencias, Universidad de Alicante, Alicante, 03080 Spain

## Abstract

During the late Pleistocene of North America (≈36,000 to 10,000 years ago), saber-toothed cats, American lions, dire wolves, and coyotes competed for prey resources at Rancho La Brea (RLB). Despite the fact that the giant short-faced bear (*Arctodus simus*) was the largest land carnivoran present in the fauna, there is no evidence that it competed with these other carnivores for prey at the site. Here, for the first time, we report carious lesions preserved in specimens of *A. simus*, recovered from RLB. Our results suggest that the population of *A. simus* from RLB was more omnivorous than the highly carnivorous populations from the Northwest. This dietary variation may be a consequence of different competitive pressures.

## Introduction

Unbalanced predator-prey densities during the Late Pleistocene of North America (≈36,000 to 10,000 years ago) resulted in more carcass encounters among large predatory mammals triggering kleptoparasitism and severe competition over kills^1–3^. As a result, saber-toothed cats (e.g., *S. fatalis*), American lions (*Panthera atrox*), dire wolves (*Canis dirus*) and coyotes (*Canis latrans*) experienced dramatic feeding stresses^1–3^, which led to a more fully and rapid consumption of carcasses (e.g., refs^4−6^).

The extraordinary fossil deposits of Rancho La Brea (RLB) tar pits in Los Angeles, California, have provided significant elements to reconstruct North American ice-age ecosystems^7,8^. RLB represents a carnivore-trap where animals got stuck on the surface of the natural asphalt seeps and attracted meat-eaters in turn^7^. Thus, the remains of large carnivores including thousands of dire wolves (*Canis dirus*), sabertoothed cats (*Smilodon fatalis*) and coyotes (*Canis latrans*)^8,9^ were preserved. Other large carnivorans less represented, include the ‘short-faced’ bear (*Arctodus simus*), the American lion (*Panthera atrox*) and the ‘scimitar-toothed’ sabertooth (*Homotherium serum*)^10^. Despite *Arctodus* being the largest land carnivoran from these ecosystems, there is no evidence that it competed with these carnivores for prey.

Here, we report the first pathological evidence in *A. simus* teeth preserved at RLB and we present a large dataset of living bear species from different North American populations affected with similar dental defects. We use macroscopic and microscopic approaches such as 3D-morphometrics of cavities from a counter mold, scanning electron microscopy (SEM), and CT analyses to ascertain the etiology of the lesions. The study confirms that unlike more northern specimens from Alaska and Yukon, dental caries were common in the population of *A. simus* from RLB, which demonstrate variable feeding preferences. Therefore, while the northern population (i.e., Alaska and Yukon) of *A. simus* was locally adapted to a highly carnivorous diet^11–13^, the population of *A. simus* from RLB was more omnivorous. We hypothesize that different competitive pressures may explain this dietary variation between both populations of this emblematic species of the North American megafauna. Moreover, this may represent evidence that the increase of the extension in the Laurentide and Cordilleran ice-sheets during the middle and late Wisconsinan isolated both populations of *Arctodus* that were adapted to feed on extremely different resources. Our findings suggest that both climatic change and local competition among ecologically interacting species are important mechanisms driving biodiversity changes at a global scale.

## Results

**Figure 1:**
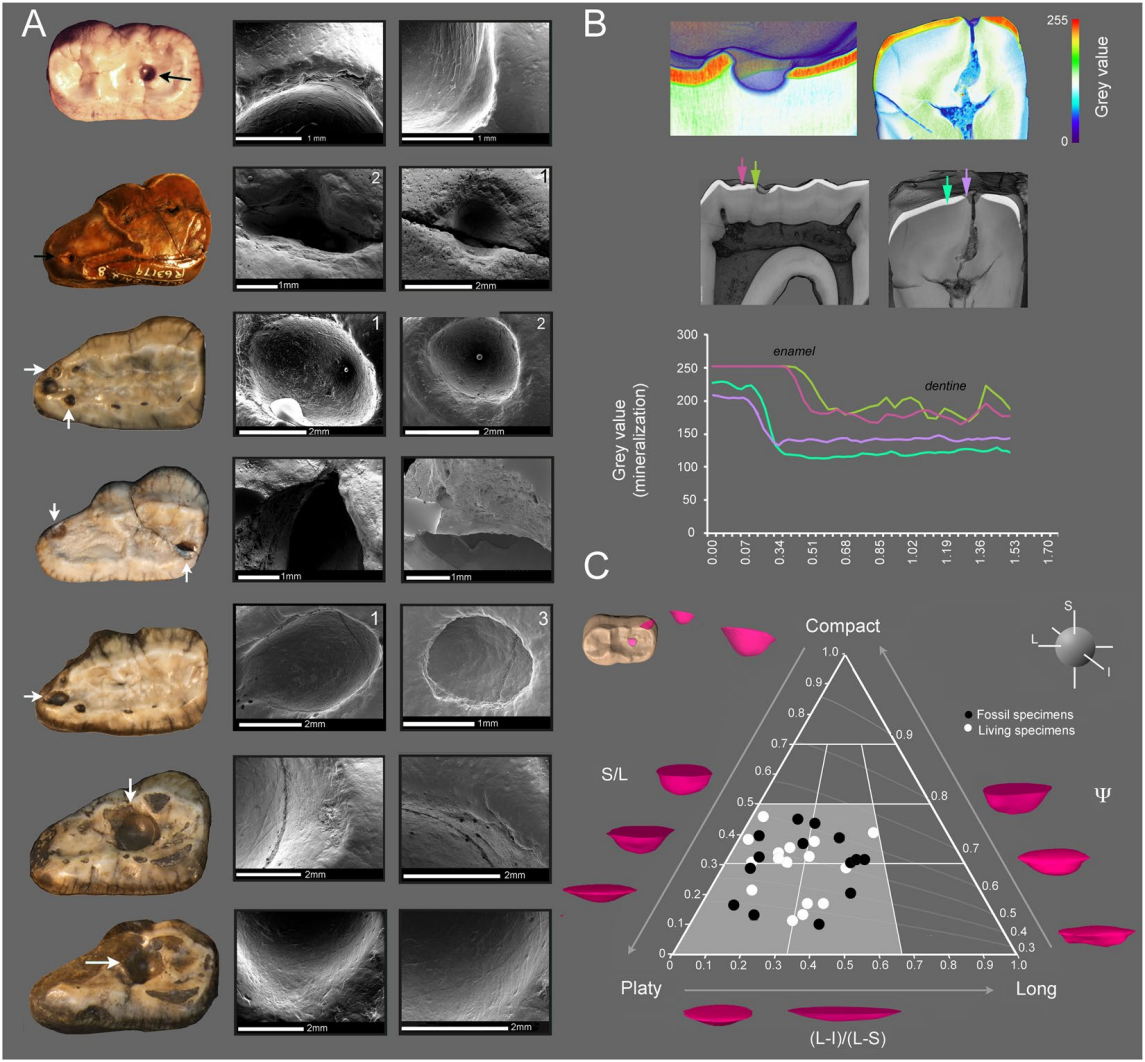
Microscopic and macroscopic analyses performed on *A. simus* pathological teeth of RLB. (**A**) Scanning Electron Microscopy (SEM) micrographs of *A. simus* teeth with carious lesions. For a complete description of fossil remains, see Supplementary Fig. S1. More details on SEM analyses are given in Supplementary Fig. S4. (**B**) Look Up Table analyses to evaluate the degree of density (mineralization) computed from CT data in LACMHC-619 (left) and LACMRLP-63179 (right). The bivariate graph shows a representation of mineralization degree across two transects sampled inside and outside the cavity (see arrows in the 3D models of above). (**C**) Ternary diagram showing size and shape of dental occlusal cavity countermolds of fossil and extant bears (see also Supplementary Table S1). For details on the three dimensional cavity countermold extraction, see Methods section. Abbreviations: S, L, I represent shortest, longest and intermediate diameters of cavity countermolds, and Ψ represents the degree of sphericity.

The 15.15% (15/33) of *A*. *simus* specimens preserved at the extraordinarily rich fossil deposits of RLB in Los Angeles (California) (*MNI* [Minimum number of individuals] = 33; *NISPs* [number of identified specimens] = 62) had pathological occlusal cavities (Fig. 1A and Supplementary Fig. S1). On the other hand, pathological occlusal cavities were not detected (0/7) in the specimens with preserved teeth from Alaska and Yukon (Supplementary Fig. S2). We also found several living bears from different North American localities affected by similar pathologies: e.g., 3.2% for brown bears (30/937) and 4.00% (45/1125) for black bears (Supplementary Fig. S3).

Furthermore, there is not any evidence of bias favoring the preservation of pathological specimens at RLB, because the ‘carnivore trap’ idea entails that carnivores were attracted by prey-dying herbivores, and the remains of *Arctodus* at RLB are substantially sparse compared to other hypercarnivores such as *Smilodon fatalis* or *Canis latrans*.

The pathologies found in *A. simus* teeth from RLB have similar locations and morphology to those observed in living bear species –i.e., in specific areas across teeth at regular intervals (Supplementary Figs S1 and S3), which differentiates *post-mortem* breakage from taphonomic processes. Based on 3D morphometric criteria (Fig. 1C and Supplementary Table S1), we identify two main groups of cavities in the teeth of extinct and extant bears. However, although both types of lesions show a continuous range, these two groups are fairly distinguishable in the Ternary diagram according to their shortest (S), longest (L), and intermediate (I) diameters of cavity countermolds, as well as to their degree of sphericity (Ψ) in Fig. 1C. Accordingly, while platy-shaped lesions form the first group, the second group is formed by blade-shaped, and mainly located on fissures of the occlusal surface. The analyzed cavities of extinct and extant specimens are morphologically indistinguishable, and show no significant differences (ANOVA; *P* < 0.05) for the intermediate (*F* = 0.578; *P* = 0.453), short (*F* = 3.148; *P* = 0.087), large (*F* = 2.817; *P* = 0.104), elongated (*F* = 0.133; *P* = 0.718) or spherical (*F* = 0.006; *P* = 0.938) cavities.

The low degree of occlusal wear present in extant and fossil teeth allows the exclusion of chipping caused by repeated attrition as a possible etiological factor^14,15^. The observation of the cavities using scanning electron microscopy (SEM) dismisses attrition as a possible etiology, as internal dentine surfaces are smooth in texture with non-chipped areas. SEM analyses (Figs 1A, S4 and S5) also allow rejecting erosion as a cause of enamel cavity formation^16^. Moreover, the cavities are located at specific points across the tooth row without any appreciable erosive action spread across the whole teeth (Fig. 1A and Supplementary Fig. S3). In addition, we did not find evidence of any extensive and uniform erosion damage of enamel and dentine consistent with acid exposure to low pH values (~0.5–2)^16,17^. Instead, SEM analysis showed regular extensive enamel micro-flake defects in the pathological hollows, and enamel texture related to tissue demineralization (Fig. 1A), which is consistent with a caries infectious etiology^18^. Indeed, this is the opposite to dental erosion, where acids originate from the diet and may thus affect the whole dentition, bacterial acids act on localized areas where biofilm is allowed to grow without disturbance and mature into an acid producing microenvironment causing carious lesions. Due to protective effects of salivary proteins, it progresses as a subsurface, rather than a surface lesion, extending their demineralization effect into dentine even before enamel breakdown^19^. Both processes are generally independent and infrequently found in the same individual^20,21^.

Otherwise, the densitometry of teeth based on high-resolution CT images computed at grey scale (look-up-table analysis or LUTs) revealed demineralization in the cavity area compared to the unaffected area (Fig. 1B). This demineralization affects enamel and dentine of the specimen LACMHC-619 and LACMRLP-R63179, where a high degree of demineralization is observed in subjacent dentine, underneath the cavity (Fig. 1B). This could be attributed to the pathology progression of the occlusal carious cavity formation, where enamel fracture creates a new biofilm retentive site over the subjacent dentin, which in turn results in further progression of dentin demineralization by harboring metabolic active cariogenic bacteria.

## Discussion

Our findings demonstrate that the population of *A. simus* from RLB regularly consumed carbohydrate-rich items, suggesting an omnivorous diet, or at least, a diet not relying solely on vertebrate flesh. Furthermore, we have found similar carious lesions across different species of living herbivorous and omnivorous bears. However, carious lesions are absent in the more flesh-eating polar bear (*U. maritimus*) (Supp. material). Although we have not found carious lesions in the bamboo-feeder giant panda (*A. melanoleuca*), they can exist in captive specimens^22^. Despite this, the giant panda has a low incidence of dental caries that could reflect the low degree of sugars that contained in the bamboo stems^23^ or the high-resistance of crenulated enamel^24^.

**Figure 2:**
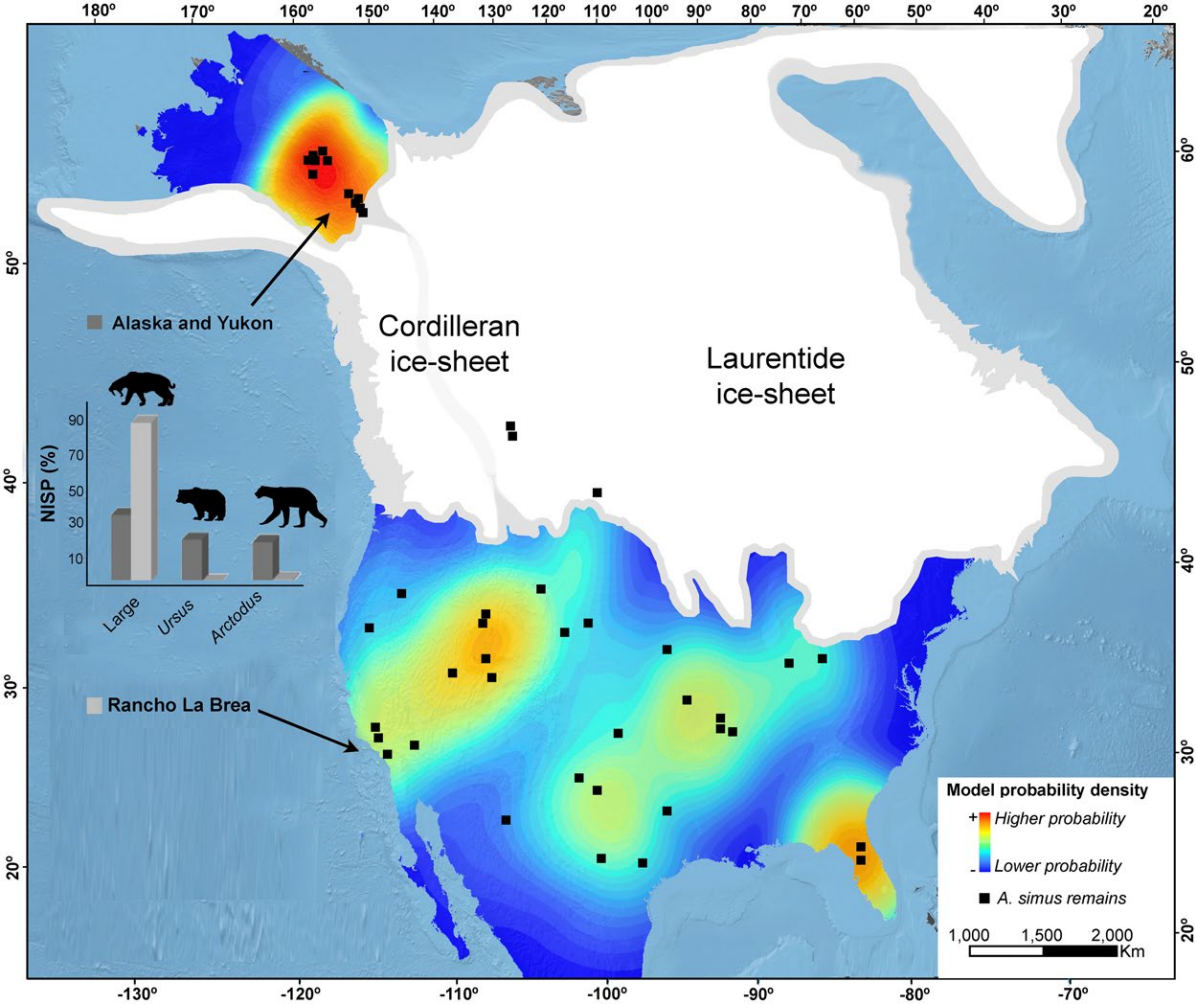
Distribution of *A. simus* in the context of intra-guild competition and climate. The North American map (i.e., excluding Mexico) is computed from a probability model based on the number of identified specimens (NISP) of *A. simus* obtained from ref.^49^. A Kernel filter for the Rancholabrean North American Land Mammal Age (NALMA) was used in ArcGis v.10.0^50^. The extension of the Cordilleran and Laurentide ice-sheets during the early late Wisconsinan (>18,000 yr BP) were drawn from ref.^51^ by B.F. Histograms represent NISP in percentage of large carnivores (*C. dirus, C. lupus, P. atrox, P. onca, H. serum, S fatalis*), *Ursus* (*U. americanus, U. arctos*) and *A. simus*. Data obtained from ref.^9,52^. While the coeval carnivores with *A. simus* in the north were *C. lupus* and *H. serum*, in the south were *C. dirus, C. lupus, P. atrox, P. onca, H. serum, and S. fatalis*. Drawings made by B.F. Note that both ice-sheets probably acted as a geographical barrier for a continuous genetic flow between the northwestern and southern populations.

The diet of *A. simus* is a contentious topic in the literature, as different researchers have proposed differing diets, including hypercarnivory relying on flesh^25–29^ and carrion^12,30–33^, omnivory^34,35^ or even herbivory^36^. Our results differ from the purely hypercarnivorous dietary interpretation of *A. simus* from RLB. On the other hand, although dental remains from the northern population are scarce, we have not detected specimens affected with similar pathologies (Fig. S2), which may indicate a non-carbohydrate (i.e., hypercarnivorous) based diet. Short-faced bears as primary predators or as scavengers are confirmed by the high proportions of δ^15^N/ δ^14^N found in bone collagen retrieved in specimens from Alaska and Yukon^11–13^. However, this population probably represents a local adaptation to feed on meat –or over the carcasses left by other carnivorans (e.g., ‘scimitar-toothed’ cat *Homotherium serum*, as proposed by others)^12,30–33^, which may explain the absence of carious lesions. The lack of the saber-toothed cat *Smilodon fatalis* from this region may imply lower levels of stress for resources in this population (Fig. 2). Given that *H. serum* was adapted to behave in more open environments and its scarce fossil record (Fig. 2), there was a low proportion of competitors and probably a lower availability of carbohydrate-rich food supplies across the year in these latitudes. In this ecological scenario, *A. simus* may have been more specialized, eating a larger proportion of meat (e.g., ref.^28^). Although *P. atrox* was also present at these latitudes in the Pleistocene, extensive radiocarbon dating suggests limited geographic and temporal overlap for *P. atrox* and *A. simus* in this region (12,990 ± 70 to 20,970 ± 180 ^14^C yr BP for *P. atrox* vs. 20,524 ± 180 to 39,565 ± 1126 ^14^C yr BP for *A. simus*)^28^. This could explain a local adaptation towards hypercarnivory in the northwest population of *Arctodus* (Fig. 2).

The ecological scenario at RLB was dramatically different, as there was a higher predator density leading to extreme intra-guild competition among large predatory mammals^1–3^, and probably a greater availability of sugary-carbohydrates. We hypothesize that this ecological situation influenced *A. simus* to behave more as a carbohydrate-feeding omnivore than as a flesh-eating (or carrion-feeder) hypercarnivore, which explains the presence of dental caries in RLB population. Therefore, our results indicate that the diet of *Arctodus* at RLB during the Pleistocene was similar to the diet of the brown (*U. arctos*) and black (*U. americanus*) bears in North America today. *Juniperus* berries^37^ or honey^38^ could be possible food resources for this population of *Arctodus*, as fossil remains for both food supplies are preserved at the site. However, as stable isotopes are useful for determining feeding preferences in fossil mammals^39^, and more particularly the case of δ^15^N/ δ^14^N because each trophic level above herbivore is indicated by an increase in δ^15^N_collagen_ between +1‰ and +6‰ (average −3.4‰)^40^, future studies on isotopic biogeochemistry (i.e., δ^15^N/ δ^14^N) could confirm (or refute) our hypothesis -based solely on caries data- about the omnivorous diet for the population of Arctodus from RLB. In either case, the dietary flexibility exhibited by *A*. *simus* in order to feed on different resources depending upon their availability compared with other large coeval carnivorans, may explain why the largest member of the carnivoran megafauna was one of the last to go extinct (10,000 yr BP)^41^, but not why it was extinct while the brown bear (*U. arctos*) persisted across North America during the ice-age^42^.

It should also be noted that the Laurentide and Cordilleran ice-sheets separated northern and southern populations of *A. simus* during part of the late Wisconsinan glaciation (Fig. 2). The impact of this separation could have limited interaction between disparate populations of *A. simus*, which may have had differing dietary strategies. Thus, we further hypothesize that the evolution of the giant short-faced bear could be a case where both abiotic (climatic change) and biotic (local competition among ecologically interacting species) factors altered the direction of lineage and/or dietary evolution.

## Methods

We examined molar teeth for the two living bears that inhabit across North America today (the black bear, *U. americanus*, *n = *1125; and the brown bear, *U. arctos*, *n* = 937), and the Pleistocene short-faced bear (*A. simus*) from Rancho La Brea (MNI = 33), and from the Northwest population of Alaska and the Yukon territory (MNI = 7), where dental remains are extremely scarce, but previous studies have demonstrated a highly carnivorous diet for these specimens^11–13^. The specimens are housed in the collections of the American Museum of Natural History of New York (USA), the Natural History Museum of London (UK), the Museum für Naturkunde of Berlin (Germany), the National Museum of Natural History of Washington DC (USA), the Canadian Museum of Nature (Ottawa, Canada) and the Yukon-Beringia Interpretative Center. We detected >75 specimens of living and extinct bears affected with dental caries lesions. Dental caries etiology was defined based on clinical features^17^ and morphological description in extant mammals, including carnivore taxa^15,43^.

### Data acquisition

High-resolution hydrophobic polyvinylsiloxane silicone-based molds were obtained from original postcanine molar crowns of those individuals with evidence of pathological conditions. The tooth crown enamel surfaces were cleaned before applying the impression material using a cotton swab soaked in 70% ethanol to remove debris and air-dried. A dual-phase technique was used to produce molds. First, a high viscosity putty soft base and their catalyzer (Virtual® Putty) were mixed and applied pressed by hand against molar teeth. When the silicone was totally set and cured (~3 min), the mold was removed and a low viscosity compound (Virtual® Light Body) was applied on the primary impression and repositioned on the specimen to increase the tooth surface accuracy and fine details resolution.

Two different types of casts were produced from tooth molds following established protocols^44,45^. First, polyurethane Feropur PR-55 (Feroca® Composites, Spain) was used to obtain non-reflective highly accurate tooth replicas optimized for further digital 3D surface models and morphological analysis^45^. A second high-resolution epoxy replica (Araldite® 2020, Vantico Ltd.) was poured for scanning electron microscopy (SEM) analyses. Epoxy-base resins are highly reliable in replicating enamel surfaces at microscopic level^45^. Two-base component epoxy or polyurethane resins were mixed and put into the molds using a Pasteur pipette. Molds were then centrifuged at 3,000 rpm during ~1 min to prevent air bubbles formation and hardener.

### Three-dimensional (3D) models

We scanned the surface of the polyurethane tooth replicas using a high-resolution NextEngine 3D laser scanner at the University of Málaga (Spain). As we already detected 72 specimens of living bears affected with dental caries, and the scanning process is highly time-consuming, we scanned a sample of 16 teeth of *U. arctos, U. americanus, U. malayanus, U tibethanus*. Later, we removed the redundant triangles, aligned different scanning views, and fusion them with Geomagic® studio. As we were interested in detecting different types of possible lesions, we compared the morphology of the cavities by constructing a diagram, commonly used in sedimentary petrology to characterize the sphericity-form for particle shapes^46^. In this diagram, the longest, shortest and intermediate diameters of each cavity countermold were calculated. Afterwards, we calculated the ratios (S/L) and (L-I/L-S) and the sphericity (Ψ = 3√ [S2/LI]) of each cavity^46^. Representing the three derived ratios, we can obtain a morphospace of countermold cavities with the compact, elongated and platy cavity shape variability. The Kolmogorov-Smirnov goodness-of-fit showed that the data comes from a normal distribution (*Z* = 0.794 to 1.113; *P* > 0.05). One-way analysis of variance (ANOVA) was computed to determine the source of significant variation among morphometric parameters. Descriptive and statistical analyses were conducted using IBM SPSS Statistics 19.0. The significant level was set at *P* < 0.05.

### Microscopic analyses

Molar teeth were examined using a scanning electron microscope (SEM) Hitachi S3000N (Servicios Técnicos Investigación, University of Alicante) for evidence of caries lesions^47^. We mounted epoxy tooth replicas on aluminum stubs with fusible glue and coated with a ~15-nm layer of gold-palladium. We applied a colloidal silver solution to improve conductivity and prevent electrostatic charges. Occlusal enamel surfaces were placed in SEM chamber perpendicular to the electron beam with zero degrees of tilt. SEM micrographs (1280 × 960 pixels in BMP file format) were recorded between 25 × and 100 × magnifications at 15Kv in secondary electron (SE) mode, and working distance (WD) ranged between 10–20 mm, depending on the size of the tooth. Microscopic taphonomic features affecting tooth-enamel and dentine tissues, which are readily identifiable were considered according to experimental reports^47^.

### CT scan

To explore patterns of enamel and dentin demineralization consistent with a carious lesion, we explored using an industrial CT scanning two selected fossil tooth specimens (LACM-HC-619; LACM-RLP-R63179; but see Fig. 1) with occlusal holes differing in shape. We used a Nikon XTH 225 ST, with acquisition conditions of 160Kv with 123µa for the first specimen, and 160Kv with 94µa for the second. For the first one, we obtained 1800 projections with a voxel size (x,y,z) of 0.042515 mm, while for the second we obtained 1800 projections with a voxel size (x,y,z) of 0.024504 mm. This information was imported to ImageJ v.1.50e (https://imagej.nih.gov/ij/) and using image filters we removed the background noise, and we fitted the range of histogram to the levels of interest R.O.I using ‘plot-profile’ to see the grey values of dentine and enamel.

We used ‘LUTs’ (Look Up Table) command from the software ImageJ to explore enamel and dentine density as a proxy for mineralization. LUT converts brightness and darkness (8-bit gray scale system where black is set to zero, and white is 255, and all of the other gradations of intensity are given values between them) in an image into a color scale that indicates the mineralization degree where the zero value was assigned to violet and 255 to red^48^. In this way, those structures with more density that reach white values correspond to red values in LUT analysis.

## Electronic supplementary material

Supplementary Information

Supplementary Data
