# Supplementary material for: Dental caries in the fossil record: a window to the evolution of dietary plasticity in an extinct bear
Source: Sci Rep. 2017 Dec 19;7:17813. doi: 10.1038/s41598-017-18116-0 (PMC5736623; doi:10.1038/s41598-017-18116-0)
Supplement: Supplementary file 1 — Supplementary Information [file 41598_2017_18116_MOESM1_ESM.pdf]

SUPPLEMENTARY INFORMATION

Dental caries in the fossil record: a window to the evolution of dietary plasticity in an extinct bear

Borja Figueirido, Alejandro Pérez-Ramos, Blaine W. Schubert, Francisco Serrano, Aisling B. Farrell, Francisco J. Pastor, Aline A. Neves & Alejandro Romero

Supplementary Figures

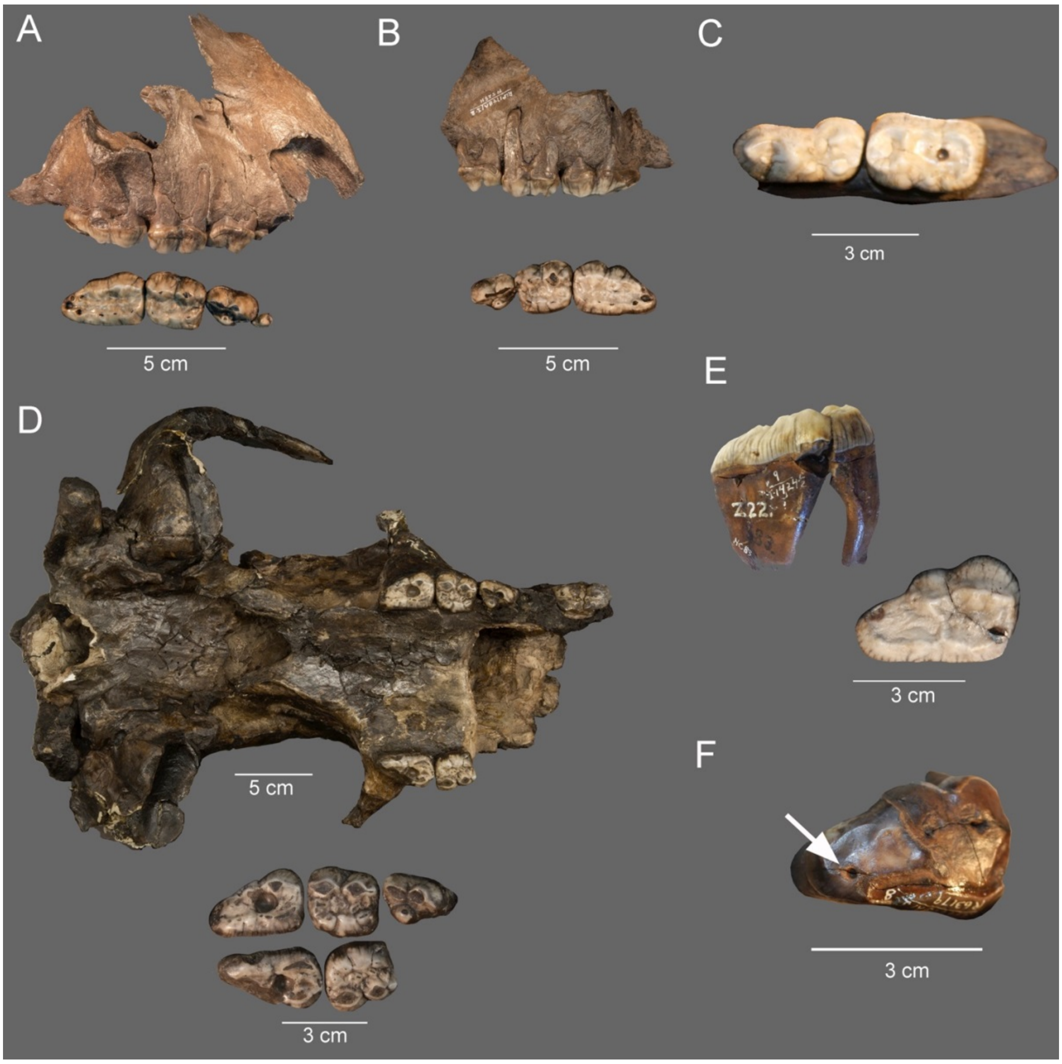

Figure S1. Fossil teeth of *A. simus* preserved at RLB affected with pathologies. (A) LACMRLP-R52237: right maxilla with preserved P3-M2; (B) LACMRLP-R52511: left maxilla with preserved P4-M2; C, LACMHC-619: right dentary with m1-m2 preserved; (D) LACMHC-Z5: partial skull with right M1-M2 and left P4-M2 preserved; (E) LACMHC-83: right isolated M2; (F) LACMRLP-R63179: right isolated M2. Note that LACMRLP-R52511and LACMRLP-R52237are both found at Pit 91, possibly representing the same individual. Pictures in A, B, D taken by Carrie Howard.

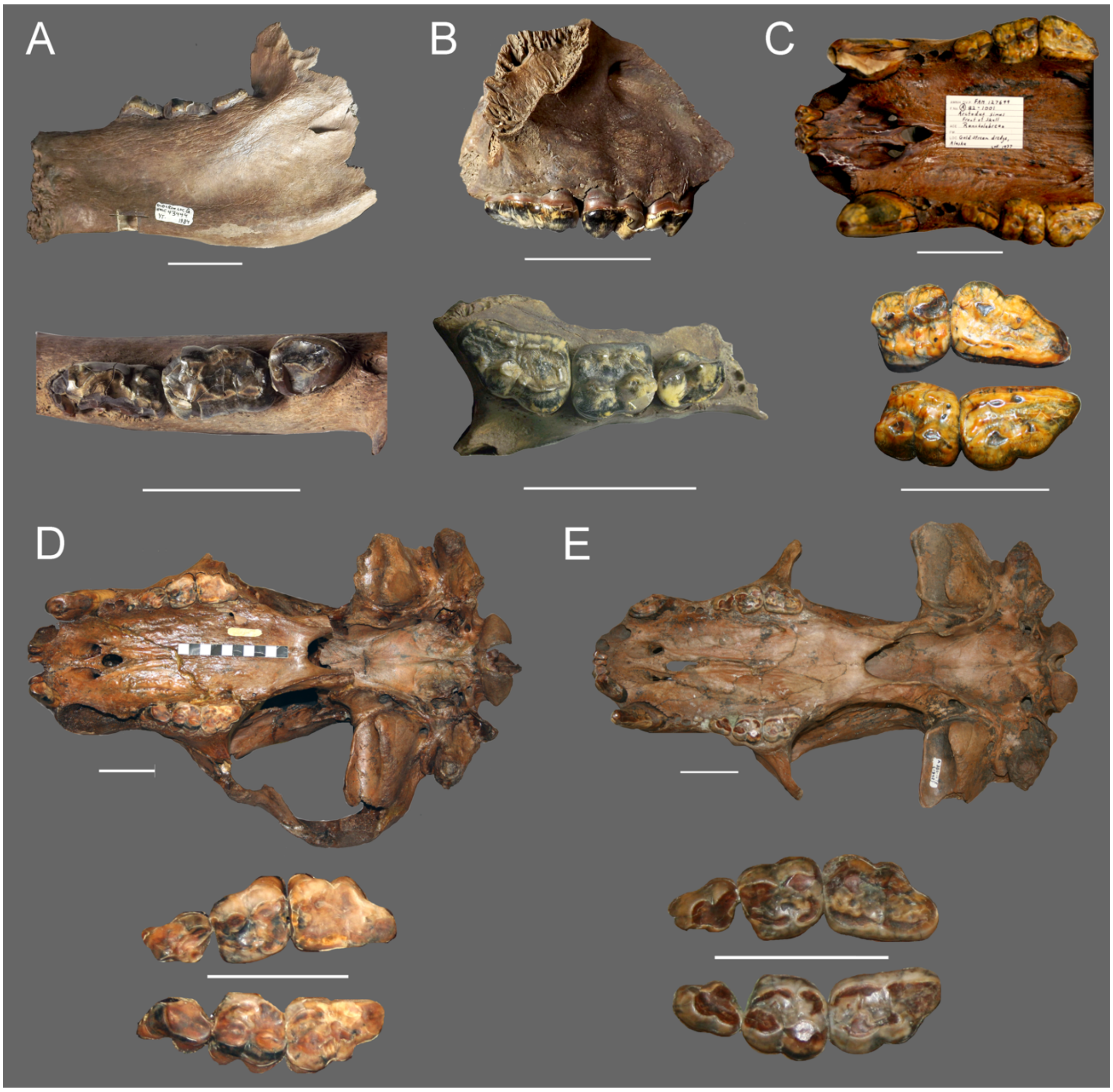

Figure S2. Fossil teeth of *A. simus* preserved at Alaska and Yukon. (A) NMC-43444: right mandible with preserved M1-M3. Old Crow (Yukon, Canada); (B) NMC-19006: right maxilla with preserved P4-M2. Old Crow (Yukon, Canada); C, F:AM-127699: right dentary with m1-m2 preserved. Goldstream (Alaska); (D) AMNH-F:AM-30492: skull with left and right dentaries. Upper Cleary (Fairbanks, Alaska); (E) AMNH: FAM-99209: skull with left and right dentaries. Ester Creek (Fairbanks, Alaska). Pictures in A, B courtesy of Dr. Danielle Fraser.

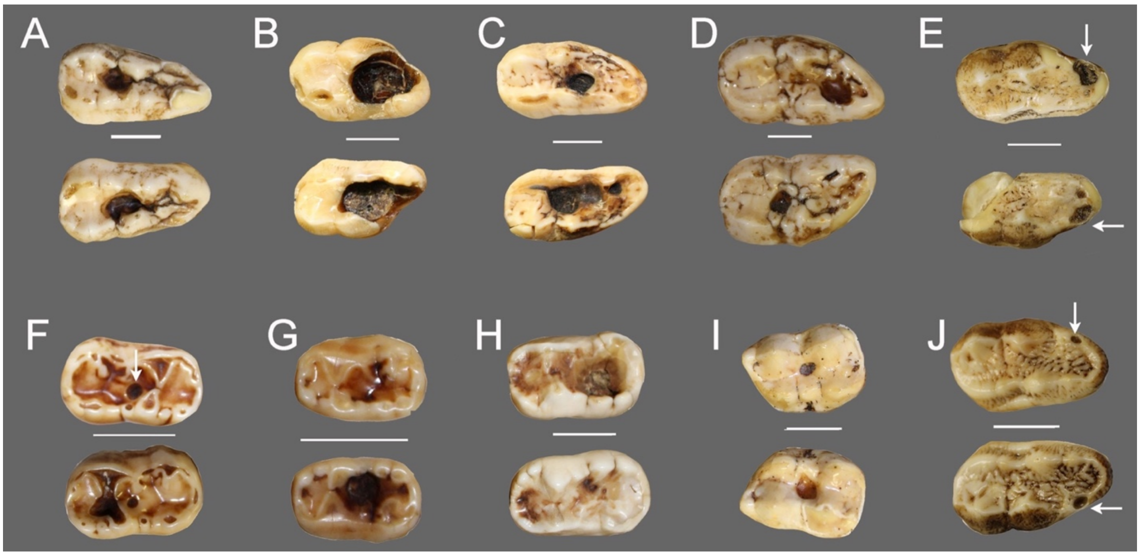

Figure S3. Selected teeth of living bears affected with pathologies. (A) *U. arctos* (USNM 205165; Yukon); (B) *U. americanus* (USNM 235458; Alaska); (C) *U. americanus* (USNM 267361; Alaska); (D) *U. arctos* (234457; Alaska); (E) *U. americanus* (USNM A21491; Nulato, Alaska); (F) *U. malayanus* (USNM 123139; Sumatra); (G) *U. malayanus* (USNM 123138; Sumatra); (H) *U. arctos* (A0441; Siberia); (I) *U. arctos* (206137; British Columbia); (J) *U. americanus* (USNM 136748; Alaska). Scale bar equals 10mm.

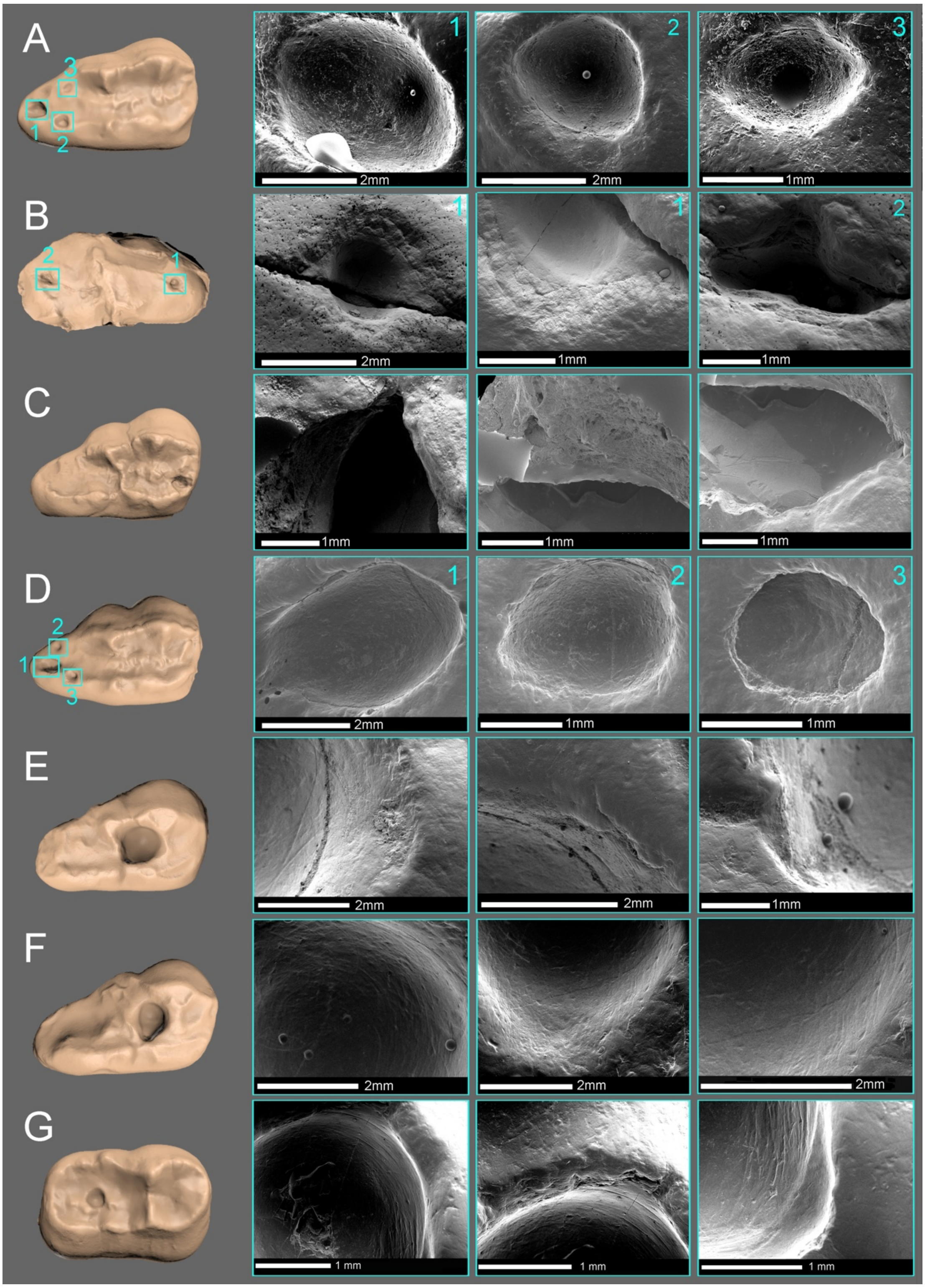

Figure S4. Scanning Electron Microscopy (SEM) micrographs of the complete sample of *A. simus* teeth preserved at RLB with carious lesions. (A) LACMRLP-R52511; (B) LACMRLP-R63179; (C) LACMHC-83; (D) LACMRLP-R52237; € LACMHC-Z5 (left); (F) LACMHC-Z5 (right); (G) LACMHC-619.

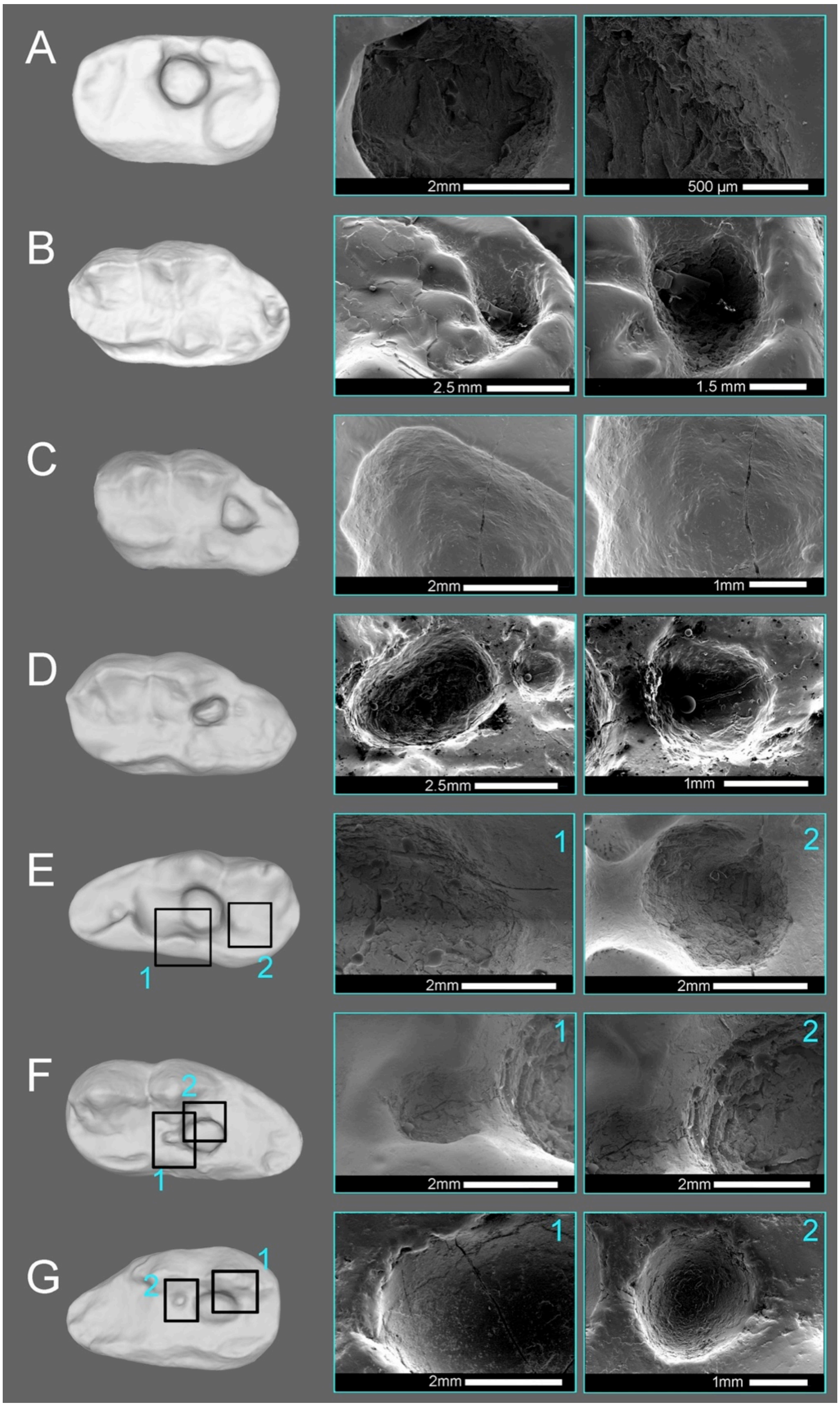

Figure S5. Scanning Electron Microscopy (SEM) micrographs of a sample of living bears with pathological teeth. (A) Lower first molar (USNM-123138) of *U. malayanus*; (B) upper second molar (USNM-074888) of *U. americanus*; (C) second upper left molar (USNM-206645) of *U. americanus*; (D) second upper left molar (USNM-231507) of *U. americanus*; (E) second upper right molar (USNM-267361) of *U. americanus*; (F) second upper left molar (USNM-267361) of *U. americanus*; (G) second upper right molar (USNM-218152) of *U. tibethanus*.

Supplementary Tables

Table S1. Morphometric variables used to characterize the shape of cavities for extant and fossil bear species. Abbreviations: I, intermediate diameter of the cavity counter mould; S, shortest diameter of the cavity counter mould; L, largest diameter of the cavity counter mould; Cl, coefficient of lengthening; Ψ = coefficient of sphericity (see methods for details). Those specimens with more than one code refer to different cavities analyzed in the same specimen.

Specimen	Code	I mm	S mm	L mm	Cl	Ψ
U. americanus	USNM-206645_1	3.42	4.32	4.43	9.41	0.41
U. americanus	USNM-206645_2	0.87	1.63	2.50	1.87	0.41
U. americanus	USNM-231507	3.31	2.54	4.07	0.50	0.16
U. americanus	USNM-155560	1.78	3.67	5.27	2.18	0.48
U. americanus	USNM-235458_1	7.51	7.74	9.87	1.11	0.27
U. americanus	USNM-235458_2	5.84	7.84	9.54	2.18	0.37
U. americanus	USNM-74888	1.87	2.21	2.64	1.80	0.33
U. americanus	USNM-267361_1	4.23	5.01	6.53	1.51	0.30
U. americanus	USNM-267361_2	4.93	6.31	7.31	2.38	0.37
U. americanus	USNM-267361_3	1.13	2.27	2.57	4.83	0.59
U. thibetanus	USNM-218152_1	3.43	3.80	5.89	1.18	0.24
U. thibetanus	USNM-218152_2	1.26	1.66	1.92	2.55	0.38
U. thibetanus	USNM-218152_3	0.68	2.11	2.97	2.68	0.74
U. arctos	USNM-234457_1	3.15	4.75	5.09	5.71	0.47
U. arctos	USNM-234457_2	1.65	4.18	6.05	2.36	0.58
U. malayanus	USNM-123138	4.14	4.40	4.53	3.07	0.34
A. simus	LACMHC-Z5_1	7.64	7.30	8.44	0.70	0.28
A. simus	LACMHC-Z5_2	6.69	6.15	7.62	0.64	0.25
A. simus	LACMHC-83	2.84	2.58	3.63	0.75	0.22
A. simus	LACMHC-619	2.71	2.98	3.69	1.37	0.30
A. simus	LACMRLP-R52237_1	1.48	2.37	2.56	5.70	0.49
A. simus	LACMRLP-R52237 _2	0.42	1.21	1.92	2.13	0.61
A. simus	LACMRLP-R52237 _3	0.44	1.43	1.73	4.28	0.90
A. simus	LACMRLP-R52237 _4	2.75	2.84	4.45	1.06	0.22
A. simus	LACMRLP-R52511_1	1.33	1.86	2.02	4.14	0.43
A. simus	LACMRLP-R52511_2	2.74	2.64	4.37	0.94	0.19
A. simus	LACMRLP-R52511_3	2.76	2.78	4.41	1.02	0.21
A. simus	LACMRLP-R52511_4	0.62	1.63	1.79	7.34	0.80
A. simus	LACMRLP-R63179_1	1.31	1.86	3.14	1.43	0.28
A. simus	LACMRLP-R63179_2	1.32	1.59	1.68	4.09	0.38
